# Cost-effectiveness of cognitive behavioural therapy for treatment-resistant depression: challenges and solutions conducting an economic evaluation of the long-term follow-up of the cobalt trial

**DOI:** 10.1186/1745-6215-16-S2-P24

**Published:** 2015-11-16

**Authors:** Kirsty Garfield, Laura Thomas, Tim Peters, Nicola Wiles, Sandra Hollinghurst

**Affiliations:** 1 University of Bristol, Bristol, UK; 2 Bristol Randomised Trials Collaboration, Bristol, UK

## Introduction

Most trial-based economic evaluations are restricted to the follow-up period of the trial. We present challenges faced conducting an economic evaluation of the long-term (LT) follow-up of a successful trial and describe solutions, which could inform similar studies.

## Methods

An economic evaluation was conducted alongside the LT follow-up of the CoBalT trial assessing the effectiveness and cost-effectiveness of cognitive behavioural therapy (CBT) for patients with treatment resistant depression.

Challenges included: follow-up at a variable interval after randomisation date; resource use data from a questionnaire smaller in scope than the trial and available for a limited period; inconsistent availability of unit costs and missing data due to loss to follow-up. Challenges were addressed by: combining questionnaire data with information from the trial to estimate average annual values of costs over the whole follow-up period; collecting detailed health care resource use over the whole period for a sample using practice notes; using a mix of inflation-adjusted and updated unit costs; and multiple imputation to estimate missing data.

## Results

The LT complete case analysis included 214 of the original 469 participants. Mean annual incremental cost to the NHS was £281; QALY gain was 0.052. The incremental cost-effectiveness ratio was £5,374. At a threshold willingness-to-pay of £20,000, this represents a 92% probability of cost-effectiveness. Results remained robust in sensitivity analyses.

## Conclusions

Despite methodological challenges, using all available information and a variety of modelling and imputation techniques, we were able to estimate annualised costs and effects of a CBT intervention over the long term.

